# Effects of Ramadan Fasting on Body Composition, Aerobic Performance and Lactate, Heart Rate and Perceptual Responses in Young Soccer Players

**DOI:** 10.2478/v10078-011-0042-9

**Published:** 2011-10-04

**Authors:** Alpay Güvenç

**Affiliations:** 1 School of Physical Education and Sports, Akdeniz University, Antalya, Turkey

**Keywords:** Ramadan fasting, body composition, aerobic exercise, soccer

## Abstract

The purpose of this study was to examine the effects of Ramadan fasting on body composition, aerobic exercise performance and blood lactate, heart rate and perceived exertion in regularly trained young soccer players. Sixteen male soccer players participated in this study. Mean age, stature, body mass and training age of the players were 17.4±1.2 years, 175.4±3.6 cm, 69.6±4.3 kg and 5.1±1.3 years, respectively. During the Ramadan period, all subjects voluntarily chose to follow the fasting guidelines and abstained from eating and drinking from sunrise to sunset. Body composition, hydration status, dietary intake and sleep duration were assessed on four occasions: before Ramadan, at the beginning of Ramadan, at the end of Ramadan and 2 weeks after the end of Ramadan. On each occasion, aerobic exercise performance and blood lactate, heart rate and rating of perceived exertion responses of players were also determined during an incremental running test. Repeated measures of ANOVA revealed that body mass, percentage of body fat, fat-free mass, hydration status, daily sleeping time and daily energy and macronutrient intake of players did not vary significantly throughout the study period (p>0.05). However, players experienced a small but significant decrease in skinfold thicknesses over the course of the study (p<0.05). Although ratings of perceived exertion at submaximal workloads increased during Ramadan (p<0.05), blood lactate and heart rate responses had decreased by the end of Ramadan (p<0.05). In line with these changes, peak running performance and running velocity at anaerobic threshold also improved by the end of Ramadan (p<0.05). Improvements in aerobic exercise performance with time were probably due to the effects of pre-season training program that was performed after the break of the fast (Iftar) during the month of Ramadan. The results of the present study suggest that if regular training regimen, body fluid balance, daily energy intake and sleep duration are maintained as before Ramadan, Ramadan fasting does not have detrimental effects on aerobic exercise performance or body composition in young soccer players.

## Introduction

Ramadan is the holiest month in the Islamic calendar and healthy adolescent and adult Muslims fast during this month. Ramadan fasting (RF) is one of the five pillars of Islam observed by over one billion Muslims worldwide. Since the Islamic calendar is based on the lunar year, which is about 11 days shorter than a solar year, Ramadan occurs at different times of the seasonal year over a 33-year cycle. During the month of Ramadan, Muslims abstain from eating, drinking, smoking and sexual activities daily between sunrise (Sahur) and sunset (Iftar). Although, there is no restriction on the amount or type of food consumed at night, food and fluid intake are exclusively nocturnal during this month. The results of previous studies in untrained subjects have indicated that food and fluid intake frequency and quantity (Leiper, 2003; Husain, 1987), nocturnal sleep duration (Roky, 2004; Margolis, 2004) and daily physical activity (Waterhouse, 2008; Afifi, 1997) are reduced during the month of Ramadan. Furthermore, dehydration (Roky, 2004; Leiper, 2003), variation in hormone levels (Bogdan, 2001), impairment in muscular performances (Bigard, 1998), increase in lipid oxidation (Ramadan, 1999) and decrease in resting metabolic rate and VO_2max_ (Sweileh, 1992) are some of the other changes observed during RF. It has been suggested that energy restriction, dehydration, sleep deprivation and circadian rhythm perturbation are possible factors influencing physical performance during Ramadan (Chaouachi, 2009b; Reilly, 2007).

Since the sporting calendar is not adapted for religious observances, and Muslim athletes continue to compete and train during the Ramadan month, it is important to determine whether this religious fast has any detrimental impact on athletic performance. However, to date, there are only a few studies concerning the effects of RF on physical performance in competitive athletes (Chaouachi, 2009a; Chennaoui, 2009; Kirkendall, 2008; Meckel, 2008; Karli, 2007; Zerguini, 2007). Many coaches and athletes still believe that athletic performance is adversely affected by RF (Chaouachi, 2009b; Leiper, 2008). But at present, there is some evidence to suggest that anaerobic exercise performance (power, speed, agility) is not negatively affected by RF in elite athletes who maintain their normal training regimen during the period of Ramadan (Chaouachi, 2009a; Kirkendall, 2008; Meckel, 2008; Karli, 2007). There are conflicting reports, however, regarding the influence of RF on aerobic exercise performance in trained athletes. A marked reduction has been reported in some studies (Chennaoui, 2009; Meckel, 2008; Zerguini, 2007), while others have found either no significant change or an increase (Chaouachi, 2009a; Kirkendall, 2008; Karli, 2007) in aerobic exercise performance during the month of Ramadan. For example, in a recent study with elite athletes, Chaouachi et al. (2009a) observed no changes either in maximal aerobic velocity or in VO_2max_ estimated from the shuttle run test during Ramadan. In another study carried out with elite soccer players, Kirkendall et al. (2008) found that the running distance during the shuttle run test improved significantly by the fourth week of Ramadan. However, in contrast to these reports, Zerguini et al. (2007) studied a group of professional soccer players and observed a marked reduction in 12-min run performance at the end of Ramadan. Inconsistent findings have also been reported with regard to the impact of RF on body composition (Chaouachi, 2009a; Chennaoui, 2009; Meckel, 2008; Maughan, 2008; Karli, 2007; Bouhlel, 2006). Nevertheless, no study to date has evaluated the effect of RF on both peak and submaximal physiological and perceptual responses during aerobic exercise in trained athletes.

On the other hand, soccer is the most popular sport in the world with more than 240 million amateur and 200 thousand professional players (Junge, 2004). It is played in all parts of the world, and participation is on the rise, especially among young non-professional players (Hoffmann, 2002). However, to date, the effects of the Ramadan fast on body composition and physical performance in young amateur soccer players have never been tested. Furthermore, although it was previously suggested that the physiological responses to exercise during Ramadan might vary depending on the fitness level of the subjects (Ramadan, 1999), all of the relevant studies were carried out with elite professional athletes.

Therefore, the purpose of the present study was to investigate the effects of RF on body composition, aerobic exercise performance and blood lactate, heart rate and rating of perceived exertion responses of regularly trained young soccer players.

## Material and Methods

### Subjects

Sixteen trained male soccer players volunteered to participate in this study. The players from the amateur soccer league comprised of five defenders, seven midfielders and four forwards. They had been training regularly for at least 3 years, with 2 hours per day and 3 days per week. Mean age, stature, body mass and training age of the players were 17.4±1.2 years, 175.4±3.6 cm, 69.6±4.3 kg and 5.1±1.3 years, respectively. Informed consent was received from subjects after a detailed description of the protocols and procedures of the study, and approval was obtained from the institutional ethics committee.

### Procedures

The study was carried out in Turkey, and all tests were completed between the months of August to October. Tests were performed during four separate sessions. Each test session was completed within 4 days. The first test session (Pre-RF) was performed during the last 4 days before the beginning of RF. The second (Beg-RF) and third (End-RF) test sessions were performed during the end of the 1st week (days 5, 6, 7 and 8) and during the last 4 days (days 26, 27, 28 and 29) of Ramadan, respectively. The fourth test session (After-RF) was conducted 2 weeks after the end of RF. During the Ramadan period, all subjects voluntarily chose to follow the fasting guidelines and abstained from eating and drinking from sunrise to sunset. For Pre-RF and After-RF conditions, subjects had their last meal at least 3 hours before the test. Tests were conducted under standard indoor conditions, where the temperature and humidity ranged from 24 to 27 °C and 52 to 57%, respectively. All tests were performed at the same time of day (between 4 and 6 pm) and in the same order to minimize the influence of diurnal variations. Subjects maintained their normal training program throughout the study but during the 24 hours before each test session, no strenuous activity was allowed. All subjects were at their pre-season training period at the time of the study. During the Ramadan phase, all players trained after the Iftar and they reported that duration, intensity and frequency of the training sessions were not reduced due to Ramadan.

### Dietary Intake, Urinary Density and Daily Sleeping Time

In this study, caloric intake, urine density and sleeping time of subjects were evaluated for each testing session (Pre-RF, Beg-RF, End-RF and After-RF). Before food intake was recorded, all subjects were informed about the analysis and were instructed on how to keep a dietary record. The records were kept for two consecutive days prior to each test session. Daily energy intake (DEI) and macronutrient proportions were assessed with nutrition information system on the computer program (BEBIS software program). The average value of the two-recorded days was reported in this study. In order to assess hydration status, each subject provided a 50 ml urine sample before each exercise test session, and then urine specific gravity (USG) was measured using a refractometer (Atago, USA). Before each testing session, subjects were also instructed to record their sleep hours for the previous two days and daily sleeping time was calculated as the mean of the two days' records.

### Anthropometry and Body Composition

Body mass (BM) was measured to the nearest 0.1 kg using a digital scale (Tanita TBF 300, Tokyo, Japan), and stature was measured to the nearest 0.1 cm using a stadiometer (Holtain, Crymych, Dyfed, UK) with subjects in light clothing and without shoes. Body mass index (BMI) was calculated by dividing BM in kilograms by the square of stature in meters. Body fat percentage (BF%), fat-free mass (FFM) and total body water (TBW) content were assessed using a foot to foot bioelectric impedance analyzer (Tanita TBF 300, Tokyo, Japan). Bioelectrical impedance measurements were performed in accordance with the manufacturer's instructions. Subjects were asked to remove all clothing (except for shorts), shoes, jewellery and other accessories for the measurement. Gender, stature and physical activity status were manually entered into the keypad interface. Subjects were measured while standing erect with bare feet on the analyzer’s footpads. Body fatness was also estimated from skinfold thicknesses. Thickness of six skinfolds (triceps, subscapular, suprailiac, abdominal, thigh and medial calf) was measured with a skinfold caliper (Holtain Ltd, UK) to the nearest 0.2 mm, on the right side of the body, using standard procedures (Lohman, 1991). Skinfold thicknesses were measured in duplicate or triplicate, according to the criteria (Lohman, 1991), and the mean of the two closest values was used for the analysis. The sum of the six skinfolds (ΣSKF) was used as an indicator of fatness. One investigator performed all the anthropometric measurements and the intraclass correlation coefficient (ICC) was greater than 0.98 for all skinfold measurements.

### Shuttle Run Test

Aerobic exercise performance was assessed using the modified 20-m shuttle run test (MSRT) which was performed on an indoor running track. After all measurements were completed, a 20-m running course with 1-m turning area behind each of the end lines, marked by plastic tape and cones, was set up in the sports hall. Following an explanation of the MSRT protocol, subjects ran back and forth between two end lines, exactly 20 m apart, in time with the audio signals emitted from a compact disc player. Each stage of the MSRT lasted 3 min with constant running pace and was separated by 1 min passive rest intervals. In the first stage of the MSRT, the initial warm-up running velocity (RV) was set at 8 km × h^−1^ then it was increased to 10 km × h^−1^ in the second stage. Thereafter, the RV was increased by 1 km × h^−1^ for each stage until the subject could not maintain the pace of the audio signals for two consecutive shuttles, or else felt fatigue and stopped running voluntarily. Before the MSRT, subjects were instructed to exert maximal effort. Subjects were also encouraged verbally throughout the MSRT to maintain the required pace as long as possible and to produce a maximal effort. Subjects wore the same running shoes and lightweight running kit for all tests. At the end of each test, the RV, the number of completed shuttles and the running time achieved at the last stage of the MSRT were recorded for each subject. Thereafter, the RV obtained at the last stage of the test was adjusted with elapsed running time and defined as the peak RV of the subjects. In addition, peak running distance (i.e. number of completed shuttles multiplied by 20 m) and peak running time (i.e. the sum of the elapsed time for each of the running periods) were calculated for each player. The MSRT was preferred to assess aerobic exercise performance, since it includes directional changes of motion and acceleration that are common during a soccer match (Eniseler, 2005). Furthermore, an indoor running track was preferred, since the environmental conditions were similar for each test session. In order to evaluate test-retest reliability and to familiarize the subjects with the testing procedures, subjects performed the MSRT twice, four days before the first test session (Pre-RF). The ICCs for peak RV, peak heart rate and blood lactate were 0.96, 0.93 and 0.88, respectively.

### Blood Lactate (LA), Heart Rate (HR) and Rating of Perceived Exertion (RPE)

Before the MSRT, at the end of each running stage (during 1 min rest intervals) and upon termination of the MSRT a 25-μL capillary blood sample was taken from the earlobe of each subject. The samples were then analyzed immediately for whole blood LA concentration by means of an electro enzymatic method using the YSI 1500 Sport Lactate Analyzer (Yellow Springs Inst., Yellow Springs, Ohio, USA). The analyzer was regularly calibrated according to the manufacturers’ instructions. By using the LA values, LA-RV curves were obtained for each subject. Thereafter, RV at a LA concentration of 4.0 mmol.L^−1^ (RV_4.0_) was calculated by using interpolation technique with a minimum R value of 0.99. The RV_4.0_ was used to display the onset of the anaerobic threshold (Janssen, 1994). HR was also recorded at 5 s intervals using HR monitors (S610i, Polar Electro Oy, Kempele, Finland) before and throughout the MSRT. Thereafter, average HR during the last minute of each 3 min running stage was calculated as the HR of that stage. In addition, RPE was assessed using the 15-point Borg scale (Borg, 1982) after the end of each running stage. The highest values of LA, HR and RPE obtained from the MSRT were used as peak responses.

### Statistical Analysis

All data were presented as mean value ± standard deviation (SD). The repeated measure of analysis of variance (ANOVA), followed by the Bonferroni correction for multiple comparisons, were used to assess the differences between Pre-RF, Beg-RF, End-RF and After-RF. Analysis of variance (two-way mixed model) was used to calculate intraclass correlation coefficients (ICCs). The alpha level of statistical significance was set at p<0.05 for all analyses. All statistical analyses were performed with the Statistical Package for the Social Sciences (SPSS Inc., Chicago, IL, USA).

## Results

As presented in Table 1, BM, BMI, FFM and TBW remained similar before, during and after RF (p>0.05). DEI, macronutrient composition of diet and DST also did not change significantly throughout the study period (p>0.05). There was little increase in USG at Beg-RF, with no significant differences being detected between test sessions (p>0.05). BF% showed a slight tendency to decrease during Ramadan, though not significantly (p>0.05). ΣSKF reduced slightly with time, and values of ΣSKF recorded at Beg-RF, End-RF and After-RF were significantly lower than that recorded at Pre-RF (p<0.05).

Peak exercise data and RV_4.0_ obtained before, during and after RF are demonstrated in Table 2. Relative to the Pre-RF values, peak RD, peak RT, peak RV and RV_4.0_ decreased slightly, though not significantly, at Beg-RF but recovered at End-RF and increased After-RF. In other words, peak running performance and RV_4.0_ recorded at Pre-RF was similar to Beg-RF and End-RF (p>0.05) but significantly lower than After-RF (p<0.05). However, peak running performance and RV_4.0_ recorded at Beg-RF was significantly lower than both End-RF and After-RF (p<0.05). Regarding peak LA, HR and RPE responses to MSRT, no significant differences were found between Pre-RF, Beg-RF, End-RF and After-RF (p>0.05).

**Figure 1:**
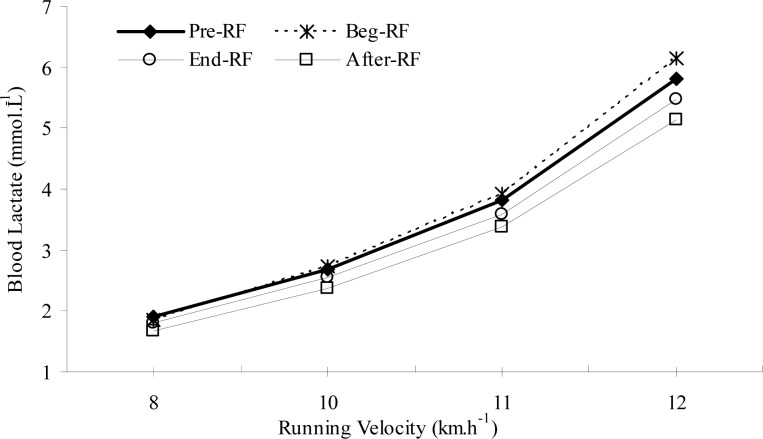
Changes in submaximal blood lactate concentrations before, during and after Ramadan fasting

**Figure 2:**
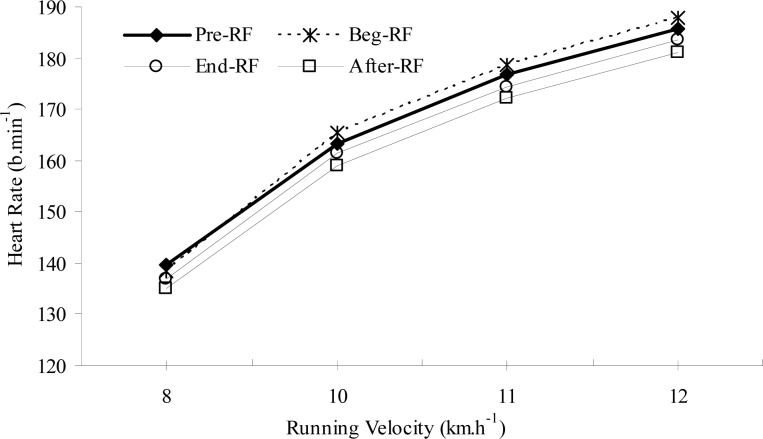
Changes in submaximal heart rate responses before, during and after Ramadan fasting

As displayed in Table 3 and Table 4, there was no significant difference between the Pre-RF, Beg-RF, End-RF and After-RF sessions for resting blood LA or HR values (p>0.05), indicating similar metabolic conditions prior to each MSRT session. Blood LA and HR responses at 8 km × h^−1^ (warm-up speed) also did not change significantly before, during and after RF (p>0.05). However, both of the responses at the higher running velocities (10, 11 and 12 km × h^−1^) were significantly lower at After-RF compared with Pre-RF and Beg-RF (p<0.05). Furthermore, during End-RF, LA and HR responses at 11 and 12 km × h^−1^ were also significantly lower than Beg-RF (p<0.05). In other words, LA-RV (Figure 1) and HR-RV (Figure 2) curves shifted to the right by the end and after RF, indicating an improvement in aerobic capacity during these periods.

**Figure 3:**
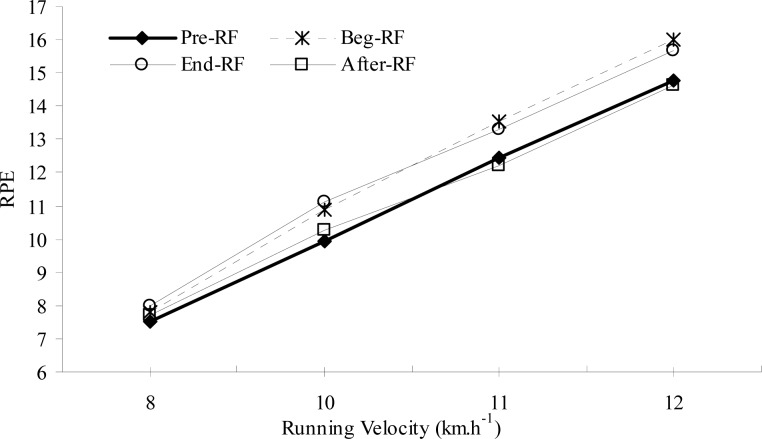
Changes in submaximal ratings of perceived exertion before, during and after Ramadan fasting

As presented in Table 5, RPE responses at 8 km × h^−1^ did not change significantly over the course of the study (p>0.05). However, relative to the Pre-RF values, RPE responses at higher running velocities (10, 11 and 12 km × h^−1^) increased significantly during Beg-RF and End-RF (p<0.05), and then returned to near Pre-RF values at After-RF (Table 5 and Figure 3).

## Discussion

The aim of this study was to examine the influence of RF on body composition, aerobic exercise performance, LA, HR and RPE responses in young soccer players who continued their usual training program. Although data on the influence of RF on physical performance are limited, this is the first study to focus on young amateur players. The main findings of the present study were that when usual training regimen, sleeping time, daily energy intake and body fluid balance were preserved through Ramadan, RF did not adversely affect aerobic exercise performance and body composition in young soccer players. Although submaximal RPE responses to aerobic exercise increased during Ramadan, objective measures of exercise intensity (LA and HR) either did not change or decreased by the end of Ramadan. Concomitant with these changes in submaximal LA and HR responses, peak running distance, peak running time, peak running velocity and running velocity at anaerobic threshold also improved by the end of Ramadan. These findings agree with other studies that have reported no detrimental effect of RF on aerobic exercise performance in regularly trained athletes (Chaouachi, 2009a; Kirkendall, 2008; Karli, 2007). However, some other studies in elite athletes reported that RF was associated with significant decreases in aerobic exercise performance (Chennaoui, 2009; Meckel, 2008; Zerguini, 2007). It was noted that the fasted athletes in these studies reduced their training loads and/or daily sleeping time during the month of Ramadan. Therefore, the findings of this study as well as those of other reports (Chaouachi, 2009a; Kirkendall, 2008; Karli, 2007) support the assumption that the athletes who maintain their training load, sleep duration, energy and body fluid balance are unlikely to suffer any substantial decline in aerobic exercise performance during the period of Ramadan.

As competitions and training programs are scheduled throughout the year, RF may present a challenge for Muslim athletes. In general, possible reductions in exercise and sporting performance during RF were attributed to daily restriction of food and fluid intake (Chaouachi, 2009b; Reilly, 2007). However, during the holy month of Ramadan, abstention from food and fluid intake start before sunrise (Sahur) and end at sunset (Iftar), and there is no restriction on the amount or type of food consumed at night. Thus, the mean daily energy and fluid intake are not necessarily reduced during this month. In the present study, energy intake values were close to those reported for professional male soccer players (13–16 MJ.day^−1^) (Burke, 2006) and did not change significantly throughout the study period. During the month of Ramadan, most of the players had night time snacks and an additional meal at night. Moreover, there was a tendency to consume foods and drinks that were higher in calories than those consumed outside of Ramadan. Thus, in accordance with previous reports (Chaouachi, 2009a; Meckel, 2008; Maughan, 2008; Karli, 2007), neither daily energy nor macronutrient intake changed significantly during Ramadan. Consistent with this result, BM, BMI, and FFM also remained similar throughout the study. The BF% tended to decrease during Ramadan, though not significantly. During the study period, the changes in BM and BF% were less than 1 kg and 1%, respectively. However, players experienced a small but significant decrease of about 2.4% in subcutaneous fat (ΣSKF) without observable changes in daily energy and macronutrient intake or lean tissue (FFM) over the course of the study. This may be related to an increased fat oxidation during Ramadan (Bouhlel, 2006) and/or the continuation of training regimen throughout the study. This may also indicate that using skinfold thicknesses from different sites may better reflect subtle changes in total body fatness.

The results of the present study are consistent with previous reports demonstrating that neither energy intake nor body composition of trained athletes changes substantially during RF. Maughan et al. (2008) found no effect of RF on daily energy intake, body fat or BMI in professional soccer players, but they did find a small reduction of approximately 0.7 kg in BM during Ramadan. Meckel et al. (2008) reported that daily energy intake, macronutrient intake and BM did not differ significantly before and after Ramadan in young elite soccer players, despite a very small change in the sum of skinfolds. Moreover, Karli et al. (2007) observed no changes either in energy intake or in body composition during Ramadan in small samples of elite power athletes. Similar results were also reported for Algerian professional soccer players by Zerguini et al. (2007), who noted no change in BM during Ramadan. In contrast to these reports and present findings, Bouhlel et al. (2006) studied a small number of elite rugby players and observed a marked reduction in energy intake (28.2%), accompanied by decreases in BM (2.2%) and body fat content (1.3%) at the end of Ramadan. Nevertheless, Chaouachi et al. (2009a) reported that energy and macronutrient intake of elite judo athletes remained similar during Ramadan but observed significant decreases in BM and body fat. Chennaoui et al. (2009), however, reported no changes either in BM or in body fat during the month of Ramadan, but did observe significant reduction in energy intake of middle-distance runners. It appears that geographical, socioeconomic, and cultural differences between Muslim countries and communities may influence the dietary practices and daily habits and thereby may contribute to the inconsistency of findings in different studies.

Sleep deprivation and dehydration during RF are other potential factors that can influence exercise performance (Chaouachi, 2009b; Roky, 2004). The present results showed that the DST did not change significantly during RF. Although there are some exceptions (Chennaoui, 2009), the results of previous studies in trained athletes are consistent with our results (Leiper, 2008; Meckel, 2008; Karli, 2007; Zerguini, 2007). Nevertheless, the present results also demonstrated that while RF did not adversely affect the objective indices of aerobic exercise performance, subjective ratings of perceived exertion at submaximal intensities increased during RF. It was noted that although there were no differences in total daily sleeping time during Ramadan, sleep and wake patterns of the fasted athletes could change (Meckel, 2008; Zerguini, 2007). Changes in sleep pattern during Ramadan have been suggested as a reason for the increase in daytime lethargy, irritability and moodiness (Reilly, 2007; Roky, 2004; Leiper, 2003). Thus, it is possible that the alternation in sleep pattern, rather than sleep deprivation, caused by RF may negatively influence RPE responses of the subjects during MSRT. However, current data showed that neither objective measures of exercise intensity (LA, HR) nor peak running performance were negatively affected by RF. Furthermore, in order to assess hydration status of players, USG and TBW measures were also obtained in the present study. Although players experienced a small increase in USG and a small decrease in TBW at the end of the first week of RF, both of the measures were not significantly changed over the course of the study. Similarly, in two recent studies with trained athletes (Shirreffs, 2008; Karli, 2007), no substantial changes in hydration status were observed over the period of Ramadan. In another study, Ramadan et al. (1999) reported significant increase in osmolarity in sedentary, but not in active subjects during Ramadan. The authors noted that body fluid balance was better maintained in active than in sedentary subjects. This might suggest that water retention mechanism of trained subjects may protect their total body water balance, which may be due, in part, to adaptation by the kidneys. It has been reported that dehydration by 2% or more of BM may impair aerobic exercise performance (Chaouachi, 2009b). In the present study, there was, nevertheless, a small weight loss of less than 1% BM during Ramadan, which was probably the result of a slight imbalance between energy intake and expenditure. However, this also may be due, in part, to acute dehydration that is known to occur throughout the hours of daylight during Ramadan (Leiper, 2003).

It has been suggested that submaximal blood LA assessment during incremental exercise is a useful tool for detecting changes in aerobic fitness, and the anaerobic threshold seems to be a better indicator of aerobic exercise performance than VO_2max_ (Edwards, 2003; Janssen, 1994). A lower LA concentration at a given submaximal exercise intensity and a higher anaerobic threshold mean that a player could maintain higher running speeds during a match without excessive accumulation of LA (Janssen, 1994). Therefore, submaximal blood LA assessment in soccer players has been previously utilized by researchers as a sensitive indicator of changes in aerobic fitness over a specified time period (McMillan, 2005; Edwards, 2003). In this study, the effects of RF on both peak and submaximal blood LA, HR and RPE responses to MSRT were examined in young soccer players. Neither resting nor peak values of LA, HR and RPE were significantly different before, during and after RF. These results indicated that subjects were tested under similar metabolic conditions and were also putting out a similar amount of maximal effort for each MSRT session. Since the MSRT includes direction changes and accelerations that are common during soccer matches (Eniseler, 2005), this field test was preferred to assess aerobic fitness. The RV_4.0_ was also calculated as an indicator of the onset of the anaerobic threshold (Janssen, 1994).

The present results demonstrated that aerobic exercise performance improved by the fourth week of Ramadan, as evidenced by a significant increase in RV_4.0_ and significant decreases in LA and HR responses at submaximal intensities during End-RF and After-RF periods. As also illustrated in Figure 1 and Figure 2, the LA-RV and the HR-RV curves shifted to the right by the end and after RF periods. In line with these changes, peak running performance improved with time. Relative to the Pre-RF value, peak running distance during the MSRT increased by about 7% at After-RF period. Moreover, peak running time increased from 15.45 min. before the beginning of RF to 16.32 min. at After-RF. In fact, a small and insignificant reduction in aerobic exercise performance was observed during the end of the first week of fasting, which then returned to, or exceeded, Pre-RF values by the end and after RF. This transient decline may suggest that there is a period of adjustment to the alternations in daily habits, lifestyle imposed by RF and training program over the first week of Ramadan.

At the time of the study, all players were at the beginning of pre-season training period. During the Ramadan phase of the study, they continued to train regularly after the Iftar. Furthermore, all players reported that they maintained their normal training program throughout the study. Indeed, probably the players returned to pre-season training in a detraining state, due to the summer break, during which they were not involved in any structured training program. Therefore, the finding of an improved aerobic exercise performance is probably mainly attributable to the training effect. It has been recognized that an adequate food and fluid intake before, during, and after training is an important means to optimize the adaptations and enhance recovery (Burke, 2006). Therefore, it appears that during the period of Ramadan, rescheduling training to other times, after the break of the fast (Iftar), is likely to be effective strategy for the fasting athletes. In addition, a previous study had shown that at the end of Ramadan, fasting led to an increase in fat oxidation during submaximal exercise in regularly trained athletes (Bouhlel, 2006). It is also possible that the increased fat utilization during the end of RF may assist exercise performance by delaying the onset of fatigue due to reducing dependence on carbohydrates as an energy source.

Consistent with current findings, in a camp setting with regularly trained professional soccer players, Kirkendall et al. (2008) found that running distance during the shuttle run test did not alter significantly in the second week of Ramadan, but by the fourth week, the results improved significantly and exceeded the pre-Ramadan values. Sweileh et al. (1992) reported a significant decrease in VO_2max_ after the first week of Ramadan, but VO_2max_ levels returned to pre-Ramadan values in the last week of Ramadan. These results may indicate a physiological adaptation to RF and/or training among the fasting subjects during the first two weeks of the fast. Furthermore, in another study carried out with elite judo athletes, Chaouachi et al. (2009a) observed no changes either in maximal aerobic velocity or in VO_2max_ estimated from the shuttle run test during Ramadan. Karli et al. (2007) also found that lactate clearance rates in elite power athletes were not affected by RF. Both Chaouachi et al. (2009a) and Karli et al. (2007) noted that the athletes in their studies did not reduce their usual training loads during Ramadan. In contrast to these results, Chennaoui et al. (2009) observed a marked reduction in maximal aerobic velocity during Ramadan in small samples of middle-distance runners. The authors suggested that the reductions in aerobic exercise performance were probably due to a lack of sleep or decreased training loads (Chennaoui, 2009). Two other studies involving elite soccer players similarly found that RF was associated with measurable decreases in aerobic exercise performance (Meckel, 2008; Zerguini, 2007). It was reported that both groups of soccer players in these two studies reduced their training loads during Ramadan (Meckel, 2008; Zerguini, 2007), which may be the main reason for these decrements in aerobic exercise performance.

## Conclusions

Collectively, these findings suggest that if regular training regimen, body fluid balance, daily energy intake and sleep duration are maintained as before Ramadan, RF does not have any detrimental effects on aerobic exercise performance or body composition in young soccer players. Although there was an increase in subjective ratings of perceived exertion at submaximal intensities during Ramadan, objective indices of aerobic performance either did not change or improved by the end of Ramadan. During the month of Ramadan, all players continued to train regularly after the Iftar. The continuation of the training program may have played an important role in this increase in aerobic capacity. Therefore, from the practical point of view, continuation of usual training program during the month of Ramadan and rescheduling training to other times, after the break of the fast (Iftar), are likely to be effective strategy for the fasting athletes and their coaches.

## Figures and Tables

**Table 1:** Body composition, hydration status, daily energy and macronutrient intake and sleeping time (means±SD) before (Pre-RF), during (Beg-RF and End-RF) and after (After-RF) Ramadan fasting (n=16)

	**Pre-RF**	**Beg-RF**	**End-RF**	**After-RF**	**F**	**p**

BM (kg)	69.56±4.29	69.19±4.39	69.00±4.93	69.52±4.66	1.14	0.342
BMI (kg/m^2^)	22.60±1.22	22.48±1.33	22.42±1.47	22.59±1.50	1.16	0.337
Body fat (%)	9.11±2.96	8.86±2.78	8.69±2.55	8.83±2.52	1.26	0.301
ΣSKF (mm)	56.30±14.55 *	55.43±14.17	55.01±14.32	54.96±13.94	10.37	0.000
FFM (kg)	63.16±3.35	63.01±3.57	62.95±4.04	63.32±3.68	0.49	0.692
TBW (L)	46.23±2.44	46.11±2.58	46.08±2.94	46.37±2.68	0.58	0.635
USG	1019.4±4.8	1021.9±4.4	1019.7±4.3	1019.1±4.9	2.05	0.120
DEI (MJ.day^−1^)	13.32±2.08	13.26±1.51	12.95±1.23	13.66±1.30	0.66	0.580
Carbohydrate (%)	52.7±7.2	50.7±7.7	51.4±7.4	53.1±7.1	2.18	0.103
Fat (%)	32.7±5.7	34.3±6.3	33.3±6.2	32.1±5.7	1.83	0.154
Protein (%)	14.6±1.9	15.1±2.0	15.3±1.7	14.8±1.8	1.30	0.285
DST (h.day^−1^)	8.6±0.5	8.8±0.5	8.7±0.7	8.6±0.4	0.50	0.684

BM: body mass, BMI: body mass index, ΣSKF: sum of the six skinfolds, FFM: fat-free mass, TBW: total body water, USG: urine specific gravity, DEI: daily energy intake, DST: daily sleeping time,

* significantly different from Beg-RF, End-RF and After-RF (p<0.05)

**Table 2:** Peak exercise data and running velocity at 4.0 mmol.L^−1^ LA concentration (means±SD) before (Pre-RF), during (Beg-RF and End-RF) and after (After-RF) Ramadan fasting (n=16)

	**Pre-RF**	**Beg-RF**	**End-RF**	**After-RF**	**F**	**p**

Peak RD (m)	2809±371 *	2638±410 #	2905±347	3009±322	10.37	0.000
Peak RT (min)	15.45±1.62 *	14.68±1.81 #	15.87±1.51	16.32±1.38	10.68	0.000
Peak RV (km.h^−1^)	14.14±0.54 *	13.89±0.61 #	14.29±0.51	14.44±0.46	10.75	0.000
RV4.0 (km.h^−1^)	11.16±0.70 *	11.11±0.55 #	11.37±0.60	11.56±0.68	8.10	0.000
Peak LA (mmol.L^−1^)	9.72±1.61	9.41±1.81	9.64±1.55	10.02±2.07	0.60	0.618
Peak HR (b.min^−1^)	192.78±7.32	193.49±7.97	191.98±7.19	192.33±7.69	0.84	0.479
Peak RPE	18.56±1.31	18.75±1.06	18.88±0.96	18.69±1.35	0.67	0.575

RD: running distance, RT: running time, RV: running velocity, RV_4.0_: running velocity at 4.0mmol.L^−1^ lactate concentration, LA: blood lactate concentration, HR: heart rate, RPE: rating of perceived exertion,

* significantly different from After-RF (p<0.05),

# significantly different from End-RF and After-RF (p<0.05)

**Table 3:** Blood lactate responses (means±SD) at submaximal running velocities before (Pre-RF), during (Beg-RF and End-RF) and after (After-RF) Ramadan fasting (n=16)

**LA responses (mmol.L^−1^)**	**Pre-RF**	**Beg-RF**	**End-RF**	**After-RF**	**F**	**p**

Resting LA	1.27±0.33	1.11±0.28	1.18±0.30	1.12±0.37	1.45	0.240
LA at 8 km.h^−1^	1.91±0.50	1.85±0.40	1.79±0.38	1.66±0.45	2.15	0.107
LA at 10 km.h^−1^	2.68±0.81	2.73±0.55 *	2.54±0.57	2.38±0.58	3.24	0.031
LA at 11 km.h^−1^	3.82±0.99 *	3.93±0.71 #	3.58±0.73	3.37±0.85	7.67	0.000
LA at 12 km.h^−1^	5.80±1.44 *	6.15±1.34 #	5.48±1.20	5.14±1.35	12.24	0.000

LA: blood lactate concentration,

* significantly different from After-RF (p<0.05),

# significantly different from End-RF and After-RF (p<0.05)

**Table 4:** Heart rate responses (means±SD) at submaximal running velocities before (Pre-RF), during (Beg-RF and End-RF) and after (After-RF) Ramadan fasting (n=16)

**HR responses (b.min^−1^)**	**Pre-RF**	**Beg-RF**	**End-RF**	**After-RF**	**F**	**p**

Resting HR	70.24±10.56	68.59±9.14	67.53±9.91	69.18±9.18	1.02	0.395
HR at 8 km.h^−1^	139.68±10.08	138.44±9.66	136.80±9.69	135.15±9.30	2.04	0.122
HR at 10 km.h^−1^	163.36±9.97	165.29±9.60 *	161.40±9.54	159.11±9.17	6.33	0.001
HR at 11 km.h^−1^	176.82±9.81 *	178.58±9.51 #	174.19±9.41	172.06±9.06	10.95	0.000
HR at 12 km.h^−1^	185.64±8.69 *	187.92±9.01 #	183.54±8.57	181.01±8.81	16.12	0.000

HR: heart rate,

* significantly different from After-RF (p<0.05),

# significantly different from End-RF and After-RF (p<0.05)

**Table 5:** Ratings of perceived exertion (means±SD) at submaximal running velocities before (Pre-RF), during (Beg-RF and End-RF) and after (After-RF) Ramadan fasting (n=16)

**RPE responses**	**Pre-RF**	**Beg-RF**	**End-RF**	**After-RF**	**F**	**p**

RPE at 8 km.h^−1^	7.50±1.21	7.81±1.33	8.00±1.10	7.69±1.40	1.16	0.334
RPE at 10 km.h^−1^	9.94±1.53 *	10.88±1.31	11.13±1.09	10.25±1.34 #	11.49	0.000
RPE at 11 km.h^−1^	12.44±1.63 *	13.56±1.41	13.31±1.20	12.19±1.64 *	16.56	0.000
RPE at 12 km.h^−1^	14.75±1.48 *	16.00±1.75	15.69±1.30	14.63±1.36 *	23.97	0.000

RPE: rating of perceived exertion,

* significantly different from Beg-RF and End-RF (p<0.05),

# significantly different from End-RF (p<0.05)
